# Implementing a Reminder System in the Northern Part of Belgium to Stimulate Postpartum Screening for Glucose Intolerance in Women with Gestational Diabetes: The “Sweet Pregnancy” Project

**DOI:** 10.1155/2017/3971914

**Published:** 2017-07-09

**Authors:** Katrien Benhalima, Sabine Verstraete, Frederik Muylle, Katelijn Decochez, Roland Devlieger, Paul Van Crombrugge, Ann Verhaegen, Johan Wens, Chantal Mathieu

**Affiliations:** ^1^Department of Endocrinology, UZ Gasthuisberg, KU Leuven, Leuven, Belgium; ^2^Diabetes Liga, Ghent, Belgium; ^3^Department of Endocrinology, AZ Jan Portaels, Vilvoorde, Belgium; ^4^Department of Obstetrics & Gynecology, UZ Gasthuisberg, KU Leuven, Leuven, Belgium; ^5^Department of Endocrinology, OLV Aalst-Asse-Ninove, Aalst, Belgium; ^6^Department of Endocrinology, AZ Jan Palfijn, Merksem, Belgium; ^7^Department of Primary and Interdisciplinary Care, University of Antwerp, Wilrijk, Belgium

## Abstract

**Aims:**

To evaluate the feasibility and efficacy of a gestational diabetes (GDM) recall register on the long-term screening uptake postpartum and to evaluate the prevalence of prediabetes postpartum.

**Methods:**

Evaluation of a GDM recall register implemented in 66 obstetrical centers in the northern part of Belgium from 2009 to 2016. Registrants receive yearly reminders to have a fasting plasma glucose test in primary care to timely detect prediabetes.

**Results:**

After 6 years, 7269 women were registered. The yearly response rates varied from 74.4% after the first year to 61.8% after the fifth year. The number of women who reported a screening test varied from 67.4% after the first year to 71.9% after the fifth year. Compared to women who responded at least once to a reminder, women who never responded were more often <30 years (41.4% versus 33.9%, *p* < 0.001) and were more often obese (29.3% versus 20.8%, *p* ≤ 0.001). Over a period of 6 years, 7.3% (CI 6.0%–8.8%) developed diabetes and 27.4% (CI 23.9%–31.0%) developed impaired fasting glycaemia.

**Conclusion:**

We show now the long-term feasibility and efficacy of a GDM recall register to stimulate screening postpartum. One-third of women developed prediabetes within 6 years.

## 1. Introduction

Gestational diabetes (GDM) has been defined as “any degree of glucose intolerance with onset or first recognition during pregnancy,” but many associations recommend now to screen for unknown diabetes at first prenatal visit (especially in women at risk) before a diagnosis of GDM can be made [1, 2]. The incidence of GDM is rising globally, and it represents an important modifiable risk factor for adverse pregnancy outcomes such as macrosomia and preeclampsia [3, 4]. Since 2013, the WHO recommends the use of the “International Association of Diabetes and Pregnancy Study Groups” (IADPSG) criteria for GDM [5]. Using the 2013 WHO criteria, GDM prevalence increases significantly in most populations and affects 9–35% of pregnancies [6, 7].

Shortly after the delivery, glucose values generally normalize again, but on the long term, women with a history of GDM have a risk that is 7 times higher compared to women without a history of GDM to develop type 2 diabetes [8]. Women with a history of GDM are also at increased risk to develop the metabolic syndrome [9]. Timely detection of glucose intolerance postpartum is important since progression to type 2 diabetes can be prevented with 50% by lifestyle intervention and/or metformin therapy [10, 11]. The American Diabetes Association (ADA) advises therefore to screen women with a history of GDM between 6 and 12 weeks after the delivery using the 2-hour 75 g OGTT [1]. The ADA further advises that these women should have lifelong screening for the development of glucose intolerance, at least every 3 years [1]. Due to the increasing prevalence of GDM, a growing number of women will need long-term follow-up postpartum to timely detect and treat glucose intolerance. This is challenging due to the often low attendance rates at screening tests postpartum and the need for long-term follow-up programs in primary care [12–14]. Many studies have reported low postpartum testing rates in routine clinical practice with only 30–50% of women with recent GDM receiving a fasting plasma glucose (FPG) or OGTT within one year after the delivery, and follow-up rates after one year are often even lower [15–19]. Small studies have shown that the use of reminders send to patients and/or health care professionals can increase the postpartum screening uptake [20]. However, very few studies have evaluated the efficacy of reminders after the first year postpartum [20, 21]. Large population-based reports are therefore needed that evaluate long-term postpartum screening programs for women with a history of GDM. Since 2009, the GDM recall register, the “Sweet Pregnancy” project, has been established in the northern part of Belgium (Flanders) [22]. This is currently the only system of long-term follow-up of women with GDM in Belgium. Women registered in the project receive annual reminders to have a FPG screening test in primary care to timely detect glucose intolerance. We evaluated the feasibility and efficacy of the GDM recall register on the long-term screening uptake postpartum in Flanders. Additionally, we also report on the prevalence of diabetes and impaired fasting glycaemia (IFG) in women registered in the recall system over a period of max. 6 years, from October 2009 until October 2016.

## 2. Methods

In October 2009, the Diabetes Liga launched the GDM recall register, the “Sweet Pregnancy” project [22]. The Diabetes Liga is an independent Flemish association representing the interests of diabetes patients and diabetes-related health care professionals. The “Sweet Pregnancy” project is an automated recall register for women with a history of GDM and is implemented in Flanders. The objective of the register is to increase the awareness among women with GDM and health care professionals on the long-term risks associated with GDM and to stimulate screening postpartum. When organizing the recall register, it was considered that the recommendation to have a yearly FPG control by the general practitioner was more practical and achievable than recommending an OGTT. An OGTT after one year postpartum was therefore not actively promoted by the project. This ongoing project is financed by the Flemish Government and is coordinated by the Diabetes Liga with the support of the Flemish association of family doctors and the Flemish association of obstetricians and gynecologists.

### 2.1. Participants

Since October 2009, the “Sweet Pregnancy” project was introduced to all 66 obstetrical centers in Flanders. Belgium has a population of 11 million of which 12% are from an ethnic minority background. Of all Belgians, 6.4 million live in Flanders [23]. The background prevalence of type 2 diabetes in Belgium is 6.5% [24]. The number of deliveries in Flanders decreases progressively over time with 63877 deliveries in 2015. The mean age at delivery in Flanders is 28.8 years, and 22.7% of pregnant women are overweight and 11.4% are obese [25].

There are currently no data on the general prevalence of GDM in Flanders. A survey performed in 2013 has shown that screening practices for GDM vary largely between centers [25, 26]. The most commonly used screening strategy for GDM in Flanders is still a two-step approach with a 50 g glucose challenge test and 100 g OGTT with the Carpenter and Coustan criteria for GDM, used by 56% of centers, followed by the one-step screening strategy with the 75 g OGTT using the 2013 WHO criteria for GDM, implemented by 25% of centers [26].

### 2.2. Registration

**Figure 1:**
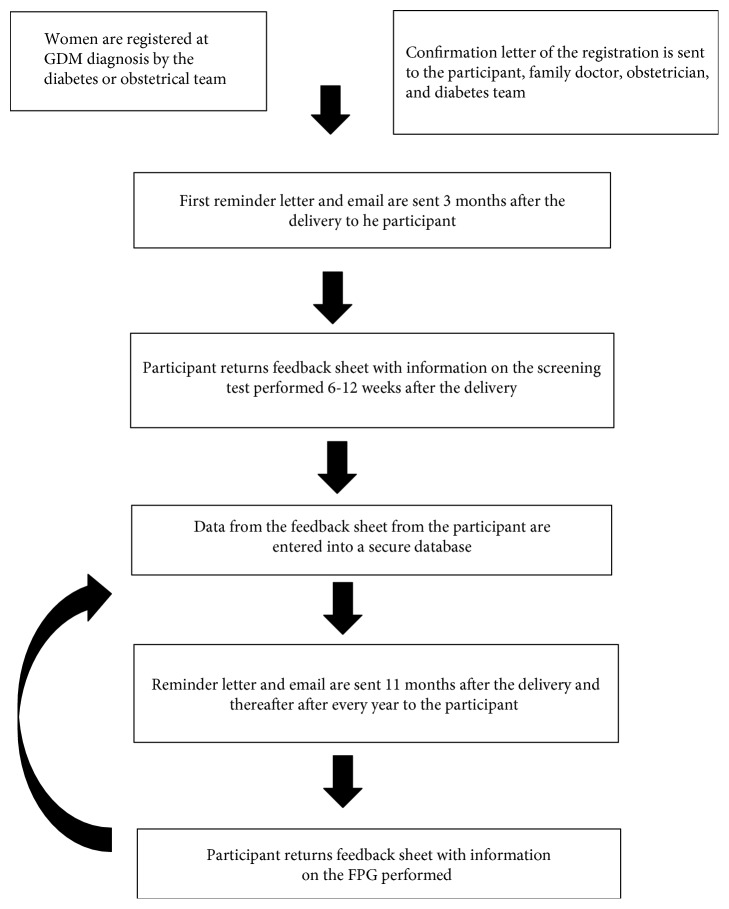
Overview of the recruitment and follow-up on the GDM recall register. GDM: gestational diabetes; FPG: fasting plasma glucose.

Figure 1 gives an overview of the recruitment and follow-up on the GDM recall register. Women with GDM are invited to voluntarily enroll on the register shortly after the diagnosis. During the recruitment, women are given an information sheet, the registration form, and a prepaid envelope. The completed registration form needs to be sent to the project coordinator of the Diabetes Liga. Since 2013, women can also register online. The registration form includes an informed consent to register participants' details and requests personal contact information, contact details of their family doctor and obstetrician, woman's date of birth, self-reported pregestational weight and length, who diagnosed GDM, and by whom and when the registration was proposed. Consent is also given on this form for the participant's general practitioner and obstetrician to be informed of their enrolment on the register. The general practitioner is asked to provide active support for the project by completing a feedback sheet. Since April 2016, the diabetes team also receives confirmation of the registration of women in the project.

### 2.3. Follow-Up

The first recall function involves sending a letter and email three months after the delivery to the registrants (Figure 1). This letter provides information on the importance of the prevention and early detection of diabetes. In addition, registrants are asked to complete a feedback sheet to check their contact details and to evaluate whether they received a screening test for diabetes 6–12 weeks postpartum. When the registrant indicates that a diagnosis of diabetes was made, a letter is sent to indicate that she will no longer receive annual reminders to perform a screening test for diabetes.

If no diabetes was diagnosed, subsequent reminder letters and emails are sent to the registrants after 11 months and yearly thereafter with the advice to visit the general practitioner to check the FPG. The registrant is asked to send the result of the FPG from each visit at the general practitioner to the coordinator of the register. If no response was obtained from the registrant after two months, an extra reminder is sent by email and/or telephone to try to get a response. Due to the large number of women registered in the project, since April 2016, telephone calls for nonresponders have been replaced by an automated SMS reminder except for the first and fifth years after the registration where a telephone call is still used. Nonresponders are defined as registrants who have never returned a response sheet to the coordinator of the register. To better promote a healthy lifestyle, since January 2016, registrants also receive 8 newsletters during the first two years of the registration with lifestyle tips for the prevention of type 2 diabetes.

The follow-up is stopped when the registrant cannot be contacted anymore, when the registrant has asked to stop the follow-up, or when the registrant has indicated that diabetes has been diagnosed. All data provided by the registrants are stored in a secure database managed by the coordinator of the project.

### 2.4. Categorization of Postpartum Glucose Testing Results

Using the ADA criteria, we defined women with a FPG < 100 mg/dl as normal, 100–125 mg/dl as IFG, and ≥126 mg/dl as having diabetes [1].

### 2.5. Feedback and Benchmarking

To evaluate the evolution of registrations and to provide feedback to the centers, an overview of the number of registrations per center is provided anonymously every 6 months to each center. The recruitment per center can thus be compared to other centers, and this provides valuable benchmarking. As part of quality control, in 2014, an online survey was sent to 500 randomly selected participants (100 women for each year of follow-up) to evaluate the perceived usefulness of the reminders to stimulate screening postpartum.

### 2.6. Statistical Analyses

The effectiveness of the GDM recall register was evaluated by analyzing the recruitment to the register, the proportion of women returning the feedback sheet (the number of responders), and the proportion of women reporting a screening test. Survival analysis techniques for time-to-event outcomes were used to analyze diabetes and prediabetes. Outcomes were defined as the time between delivery and the first assessment of prediabetes. The cumulative risk of diabetes was estimated using the Kaplan-Meier method, whereas for prediabetes estimation was based on the cumulative incidence function. Diabetes without earlier prediabetes was then considered as a competing risk. Patients without prediabetes were censored at the last follow-up. The independent contribution of participant characteristics was analyzed using Cox proportional hazard models, competing risks being censored. The results are presented as hazard ratios (HR) with 95% confidence intervals. The chi-square test was used to test the association between responder/nonresponder and participant characteristics. A *p* value of <0.05 (two tailed) was considered significant unless stated otherwise. Analyses were performed using SAS software (version 9.4).

## 3. Results

### 3.1. The Number of Registrations over Time and per Center

**Figure 2:**
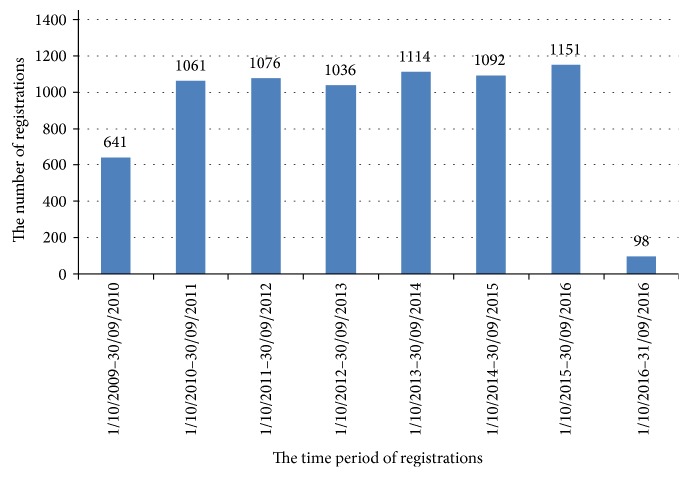
Overview of the number of registrations per year in the register over the 7-year period.

Since the start of the register in October 2009, 7269 women have registered. Yearly, more than 1000 women have registered in the register (Figure 2()). Participation in the register was generally similar across all provinces, with the highest rates of registration in West Flanders and Limburg with, respectively, 138 and 136 registrants per 100,000 inhabitants, followed by Flemish Brabant and Antwerp with, respectively, 116 and 105 registrations per 100,000 inhabitants. The lowest number of registration was seen in the province of East Flanders with 71 registrations per 100,000 inhabitants. Of all 66 centers, 35% (23) had more than 100 registrations. Data on length and weight of the registrants were reported in the feedback sheet by 97.2% of all registrants, and 94.3% of all registrants provided contact details. The mean age of the registrants was 32 ± 4.8 years, 27.9% were overweight, and 23.2% were obese. Of all obstetricians in Flanders, 76% (511) came in contact with the project. Of all general practitioners in Flanders, 39.3% (3323) came in contact with the project and 69.4% (2307) of them indicated their active support for the project.

### 3.2. The Number of Responses and Screening Tests over Time

Of all registrants, 84.4% (5465) responded to the letter sent three months after the delivery and 58.8% (3215) of responders indicated that they had received a screening test to detect glucose intolerance in early postpartum and 2.8% (91) reported to have diabetes based on the early postpartum screening test.  Table 1 gives an overview of the number of responses and the number of responders who received a screening test by each year of follow-up. The yearly response rates varied from 74.4% after the first year to 61.8% after the fifth year. The primary response rate before an extra reminder was sent was 23.3% after the first year and 22.0% after the fifth year. Of all responders, 67.4% reported a screening test after the first year and 71.9% after the fifth year.  Table 2 gives an overview of the number of responses and screening tests of all registrants who delivered before October 2011 and received a yearly reminder during 5 consecutive years. Of all women who received a yearly follow-up letter (1157) and were 5 years in follow-up in the register, 75.0% (868) received at least once a screening test, 60.6% (701) received at least twice a screening test, 46.6% (539) received at least three times a screening test, 34.0% (393) received at least four times a screening test, and 18.3% (212) of women received yearly a screening test over the 5-year period.

### 3.3. Survey among Registrants to Evaluate the Perceived Usefulness of the Reminders

The survey had a response rate of 26.4% (132), and 70.5% (93) of responders indicated that the reminders were important to stimulate screening and that without the reminder, they would not have visited their general practitioner for a screening test.

### 3.4. The Characteristics of Nonresponders Compared to Responders

Compared to women who responded at least once to a reminder, women who never responded were more often <30 years (41.1% versus 33.9%, *p* < 0.001) and were more often obese (29.3% versus 20.8%, *p* = <0.001).

### 3.5. The Number of Responders with (Pre)diabetes over Time

**Figure 3:**
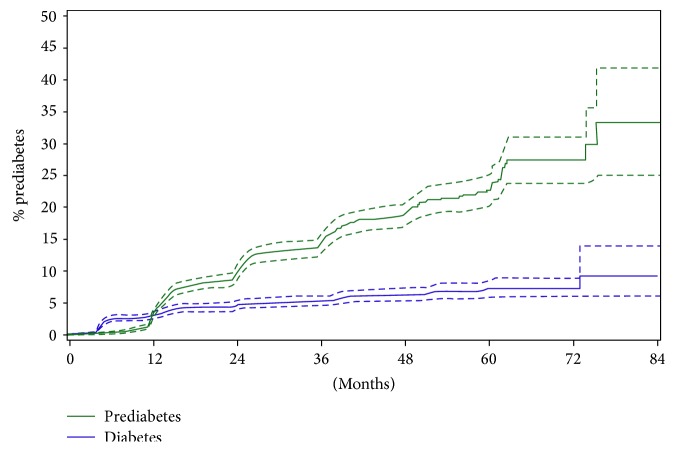
Cumulative risk of diabetes and prediabetes over time since the delivery (+95% CI). The crossing of the two curves is the result of data registration: diabetes may have been detected and registered at 3-month postnatal screening, hence the “bump” around 3 months in the curve for diabetes. Prediabetes was only registered at long-term yearly follow-up with the GP, hence the “bump” around 12 months (and with 12-month intervals thereafter).

The cumulative risk after 1 year for diabetes and IFG was, respectively, 3.1% (CI 2.6%–3.7%) and 3.4% (CI 2.9%–4.1%). After 3 years, the cumulative risk for diabetes and IFG was, respectively, 5.2% (CI 4.5%–6.1%) and 14.5% (CI 13.1%–16.0%) and after 6 years, the cumulative risk for diabetes and IFG increased to, respectively, 7.3% (CI 6.0%–8.8%) and 27.4% (CI 23.9%–31.0%) (Figure 3).

### 3.6. Predictors for Prediabetes

In the univariable analyzes, predictors for diabetes and prediabetes were increasing age, BMI, and waist circumference, and for diabetes also the fact that women received a screening test within 3 months after the delivery (Table 3(a)). In the multivariable analyzes, age and BMI remained independent predictors for diabetes and prediabetes and for prediabetes, waist circumference was also an independent predictor (Table 3(b)).

## 4. Discussion

Although there is a general consensus that timely detection and treatment of glucose intolerance after the delivery in women with GDM is important, postpartum testing rates in normal routine are often very low [15–19]. In our register, of all responders, only 58.8% indicated that they received a screening test within three months after the delivery. This is a missed opportunity to timely identify and treat high-risk women for glucose intolerance. Small studies have shown that the use of reminders sent to patients and/or health professionals can increase the postpartum screening uptake [20]. However, very few studies have evaluated the efficacy of reminders after the first year postpartum [20, 21]. We show now the feasibility and efficacy of a GDM recall register implemented across 66 centers in Flanders (Belgium). The response rate was the highest after the first year of follow-up but after 5 years, nearly 62% still responded to the reminder. The screening rates remained generally stable over the years and varied from 62.1% to 71.9%. We provide now the first data of the successful use of a GDM recall register on the long term to stimulate screening postpartum. Moreover, we show now that the register is widely implemented in a large region as part of normal routine care. However, it remains challenging to stimulate women to get a yearly screening test since only 18.3% of women with 5 years of follow-up in the register reported a screening test every year.

A recent systematic review including 6 studies has shown that both reminder systems send to patients and/or health care professionals are successful in increasing postpartum screening rates with a glucose test performed in 50–71% of women. However, the evidence is very limited for long-term effects past the first year postpartum [20]. Reminders by telephone seem to be more effective than letters, and with more frequent reminders, more women will undergo a follow-up visit over time [20]. The response rate to the reminders was markedly higher (between 60.2% and 74.4%) in our register compared to the South Australian GDM recall register where 68.4% of women with GDM registered but only 46.4% returned the update form [21]. In the South Australian GDM recall register, women who did not respond to a reminder letter were not followed up for a response while our register did not only use annual reminders by letters and email but if needed, additional email, telephone, or SMS reminders were used to increase the response rate [21]. By using extra reminders, the response rates increased importantly in our register. There is currently only one RCT in women with GDM that evaluated the use of reminders to increase screening with an OGTT within 1 year after the delivery. The study showed that postal reminders send to patients or physicians alone or send to both patients and physicians increased screening rates from 14.3% to 51.6%–60.5% [27]. The highest screening rate postpartum (60.5%) was seen in the combined patient/physician group [27]. However, when this reminder system was implemented into routine care, screening rates postpartum only increased to 28% [28]. Although we have no data on the long-term screening uptake postpartum before the use of the register in Belgium, many studies have shown a long-term screening uptake of max. 20% in normal routine [18, 19]. A survey among participants has shown that for many women, the reminders were perceived very useful to stimulate screening. Our data highlight therefore that our GDM recall register implemented in routine clinical care is feasible with sustained high registration and response rates.

Age and BMI of registrants were similar as previously reported in women with GDM from two large Flemish hospitals, but the register does not have any data on ethnicity or the socioeconomic status of the participants [29]. Compared to women who responded at least once to a reminder, women who never responded to a reminder in our register were more often <30 years and were more often obese. Younger women may underestimate their risk to develop glucose intolerance while women with a higher risk profile such as obese women are often difficult to engage in screening programs [30, 31]. It has been shown that the nonattendance rate at the postpartum OGTT is particularly high in women from an ethnic minority background and women with an adverse metabolic profile [30, 31]. These women have often the highest risk to develop glucose intolerance postpartum, and more efforts are clearly needed to stimulate these women to attend the postpartum screening tests [31]. Important barriers to postpartum screening are the lack of patient attendance, most often due to time pressure, clinicians' perception that screening guidelines are inconsistent, lack of documentation of GDM in the medical file, and poor communication between obstetricians and primary care providers [12–14, 32]. Studies have shown that predictors of higher screening rates include older age, nulliparity, and higher income and education. Women who received prenatal care, women who needed insulin during pregnancy or who came to the 6-week postpartum visit, were also more likely to receive screening [13, 14, 32]. This might explain why women who received a screening test in early postpartum in our register were more likely to develop diabetes over time as women who adhere better to early postpartum screening might also adhere better to follow-up screening.

Prevalence of glucose intolerance after the delivery in women with GDM varies according to the population studied and how women with GDM were identified. We have previously shown that in a large university center in Flanders, about 44% of women with GDM diagnosed by a two-step approach with the Carpenter and Coustan criteria or the 2013 WHO criteria for GDM had glucose intolerance based on the 75 g OGTT three months after the delivery [30, 31]. On the long term, studies have shown that 16–50% of women with GDM develop type 2 diabetes [8, 13, 33, 34]. Our data show now that over a period of 6 years, 27.4% of all responders developed IFG and 7.3% developed diabetes.

The Flemish guideline for the diagnosis and treatment of type 2 diabetes recommends a FPG as screening test instead of an OGTT because this is more feasible to perform in primary care [35]. The “Sweet Pregnancy” project chose therefore a practical approach for follow-up in primary care with a FPG as screening test. The FPG may be more acceptable to women because it requires less time, which in turn might increase the attendance rates at screening tests and increase the long-term follow-up postpartum. However, many studies have shown that by only using a FPG, a substantial number of women can be missed with diabetes or impaired glucose tolerance [13, 17, 30, 31]. Moreover, a simulation showed that when annual, biannual, or every 3-year screening strategies were utilized, OGTTs resulted in lower costs per case detected than FPG or HbA_1c_ [36]. It is therefore very likely that the currently reported prevalence of glucose intolerance with the register is an underestimation. The ADA recommends lifelong screening for the development of glucose intolerance, at least every 3 years. When glucose intolerance is found, the frequency of screening should be increased to at least annually [37]. However, the ADA makes no clear recommendation as to which test should be used (HbA_1c_, FPG, or 2 h 75 g OGTT) since the ADA considers that there is insufficient evidence to recommend one test over the other [1]. Studies evaluating the use of HbA_1c_ alone or in combination with FPG to diagnose glucose intolerance in women who have had GDM show conflicting results with sensitivity rates of HbA_1c_ and FPG combined ranging from 83.0% to 90.0% [38, 39]. We have no data on HbA_1c_ in our register.

The most important risk factors to develop glucose intolerance in early postpartum differ according to the populations studied [40]. Maternal age, prepregnancy weight, early GDM diagnosis, pharmaceutical treatment during pregnancy, and the FPG on the diagnostic OGTT during pregnancy often emerge as important risk factors for glucose intolerance postpartum [17, 40, 41]. This is in line with our data showing that women with a higher age, BMI, and waist circumference had the highest risk for glucose intolerance postpartum.

Strengths of our register are the large number of participating centers across a large region with a long follow-up. The recall register is widely implemented, and nonresponders receive systematically a new reminder by email, telephone, or SMS. Limitations are the limited clinical data in the register, and the data obtained are self-reported by the registrants. Self-reporting of screening tests might have overestimated the screening uptake postpartum due to selection of the most engaged women [32]. Due to the lack of uniformity on screening for GDM in Flanders, there are differences between centers in how women with GDM were diagnosed. We could also not evaluate whether the missed tests are due to lack of order by the doctor or because women did not visit the doctor. From the group of registrants who did not respond, we have no information on whether or not they received a screening test. As we have no data on women who were not offered registration or did not want to register, we do not have a control group to compare the efficacy of reminders with on screening uptake postpartum. For future research, the database should be extended with data on the socioeconomic status and the ethnicity of registrants. Linking the register to the general electronic health record will be an important step to collect and validate more data on the registrants.

In conclusion, we show now the long-term feasibility and efficacy of a GDM recall register implemented as part of normal routine in a large region to stimulate screening postpartum. One-third of women developed prediabetes within 6 years based on the FPG. However, more research is necessary to better understand how to engage and stimulate high-risk women to respond.

## Figures and Tables

**Table 1:** An overview of the number of responders and screening tests per year of follow-up.

	1 year of follow-up	2 years of follow-up	3 years of follow-up	4 years of follow-up	5 years of follow-up
The number of yearly reminders sent	4765	3561	2482	1557	542
Number of responders (%)	3547 (74.4%)	2419 (67.9%)	1542 (62.1%)	877 (60.2%)	335 (61.8%)
Number of responders who received a screening test (%)	2390 (67.4%)	1502 (62.1%)	1060 (68.7%)	612 (69.8%)	241 (71.9%)
Number with diabetes	104	26	15	3	0

The number of responders is the number of registrants who returned a response sheet. The number of responders who received a screening test is the number of responders who have indicated that they received a screening test in primary care in the past year. The number with diabetes is based on self-reporting of the test between 6 and 12 weeks postpartum and based on self-reporting of the screening tests received after 1 year.

**Table 2:** An overview of the number of responders and screening tests of all registrants who received a yearly reminder during 5 consecutive years.

	Follow-up year 1	Follow-up year 2	Follow-up year 3	Follow-up year 4	Follow-up year 5
Number of women who received a follow-up letter	1370	1202	1273	1221	1157
Number of responders (%)	1128 (82.3%)	913 (76.0%)	848 (66.6%)	737 (60.4%)	686 (59.3%)
Number of responders who received a screening test (%)	748 (66.3%)	586 (64.2%)	575 (67.8%)	524 (71.1%)	489 (71.3%)

The number of responders is the number of registrants who returned a response sheet. The number of responders who received a screening test is the number of responders who have indicated that they received a screening test in primary care in the past year.

**Table:** (a) Results of univariable analyses

Variable	Contrast	Diabetes	Diabetes *p* value		Prediabetes	Prediabetes *p* value	
		Hazard ratio (95% CI)			Hazard ratio (95% CI)		
			Contrast	Global		Contrast	Global
Age category (years)	30–40 versus <30	1.40 (0.98 : 2.00)	0.0646	0.0032	1.10 (0.88 : 1.36)	0.4049	<0.0001
	>40 versus <30	2.53 (1.48 : 4.33)	0.0007	—	2.08 (1.47 : 2.93)	<0.0001	—
	>40 versus 30–40	1.81 (1.12 : 2.91)	0.0151	—	1.90 (1.39 : 2.60)	<0.0001	—
BMI category (kg/m^2^)	25–30 versus <25	1.83 (1.28 : 2.61)	0.0009	<0.0001	1.94 (1.54 : 2.44)	<0.0001	<0.0001
	>30 versus <25	2.33 (1.61 : 3.38)	<0.0001	—	2.77 (2.20 : 3.48)	<0.0001	—
	>30 versus 25–30	1.28 (0.88 : 1.86)	0.2042	—	1.43 (1.13 : 1.81)	0.0032	—
WC category (cm)	80–88 versus <80	0.63 (0.14 : 2.94)	0.5594	0.0462	1.01 (0.68 : 1.50)	0.9515	<0.0001
	>88 versus <80	2.47 (0.79 : 7.74)	0.1208	—	2.39 (1.76 : 3.23)	<0.0001	—
	>88 versus 80–88	3.90 (1.14 : 13.39)	0.0304	—	2.36 (1.70 : 3.26)	<0.0001	—
Support GP	Yes	1.09 (0.74 : 1.62)	—	0.6617	1.23 (0.96 : 1.57)	—	0.1076
Received screening in early postpartum	Yes	3.07 (2.18 : 4.31)	—	<0.0001	1.06 (0.88 : 1.27)	—	0.5363

WC: waist circumference; support GP: general practitioner confirmed active support for the recall register; received screening in early postpartum: between 6 and 12 weeks after the delivery; HR: hazard ratio; CI: confidence interval; *p* values: global for the variable as a whole; contrast for difference between 2 categories; HR > (<)1 means higher (lower) risk for the first category.

**Table:** (b) Results of multivariable model

Variable	Contrast	Diabetes	Diabetes *p* value		Prediabetes	Prediabetes *p* value	
		Hazard ratio (95% CI)			Hazard ratio (95% CI)		
			Contrast	Global		Contrast	Global
Age category (years)	30–40 versus <30	2.41 (0.67 : 8.69)	0.1799	0.0195	1.18 (0.89 : 1.58)	0.2506	0.0388
	>40 versus <30	6.75 (1.64 : 27.85)	0.0082	—	1.84 (1.15 : 2.93)	0.0108	—
	>40 versus 30–40	2.81 (1.06 : 7.41)	0.0373	—	1.55 (1.02 : 2.36)	0.0396	—
BMI category (kg/m^2^)	25–30 versus <25	6.01 (2.42 : 14.92)	0.0001	<0.0001	1.55 (1.09 : 2.21)	0.0145	0.0001
	>30 versus <25	8.19 (2.92 : 23.01)	<0.0001	—	2.30 (1.57 : 3.37)	<0.0001	—
	>30 versus 25–30	1.36 (0.56 : 3.31)	0.4935	—	1.48 (1.08 : 2.03)	0.0152	—
WC category (cm)	80–88 versus <80	0.37 (0.10 : 1.32)	0.1252	0.2203	0.88 (0.59 : 1.32)	0.5414	0.0380
	>88 versus <80	0.59 (0.25 : 1.38)	0.2220	—	1.41 (0.95 : 2.11)	0.0906	—
	>88 versus 80–88	1.61 (0.43 : 6.06)	0.4822	—	1.61 (1.10 : 2.34)	0.0133	—

WC: waist circumference; HR: hazard ratio; CI: confidence interval; *p* values: global for the variable as a whole; contrast for difference between 2 categories; HR > (<)1 means higher (lower) risk for the first category.
